# Evaluation of Trabecular Bone Microarchitecture for Zygomatic Implants: A Cone Beam Computed Tomography Study

**DOI:** 10.3390/diagnostics16213456

**Published:** 2026-10-23

**Authors:** Emre Karahan, Simge Eşme, İrem Öztürk, Burak Bilecenoğlu, Seçil Aksoy, Kaan Orhan

**Affiliations:** 1 Department of Dentomaxillofacial Radiology, Faculty of Dentistry, Ankara University, Ankara 06560, Türkiye; irmozturk@ankara.edu.tr (İ.Ö.); call53@yahoo.com or knorhan@dentistry.ankara.edu.tr (K.O.); 2 Department of Anatomy, Faculty of Medicine, Ankara Medipol University, Altındağ, Ankara 06050, Türkiye; simge.esme@ankaramedipol.edu.tr (S.E.); burak.bilecenoglu@ankaramedipol.edu.tr (B.B.); 3 Department of Dentomaxillofacial Radiology, Faculty of Dentistry, Near East University, Mersin 99138, Türkiye; dtsecilgunay@yahoo.com.tr; 4 Ankara University Medical Design Application and Research Center (MEDITAM), Ankara 06100, Türkiye; 5 Department of Oral Radiology, Semmelweis University, 1088 Budapest, Hungary

**Keywords:** zygomatic implants, trabecular bone, cone beam computed tomography, microarchitecture

## Abstract

**Background/Objectives**: Zygomatic implants are commonly used to rehabilitate atrophic maxillae and maxillary defects, offering quicker prosthesis placement than bone grafting, but their placement requires technical precision due to the complexity of the zygomatic region. Trabecular bone quality is a key determinant of implant stability, yet data on the zygomatic bone’s microarchitecture remain limited. This study aimed to evaluate and compare trabecular bone microarchitectural features of the zygomatic bone with three reference sites (first molar region, mandibular condyle, and angle of mandible) using cone beam computed tomography (CBCT). **Methods**: This retrospective cross-sectional study screened 847 archived CBCT scans from the Ankara University Faculty of Dentistry, of which 99 systemically healthy patients aged 20–30 years were included. Trabecular bone microstructural parameters, including tissue volume, bone volume, percent bone volume, trabecular thickness, trabecular number, trabecular separation, and fractal dimension, were assessed using CTAn software at the zygomatic bone, first molar region, mandibular condyle, and angle of mandible. **Results**: The zygomatic bone exhibited significantly higher tissue and bone volumes than the other regions but lower percent bone volume and trabecular number; trabecular thickness and separation were greatest in the zygomatic bone, while fractal dimension values were lowest. When pooled across the four regions, significant right–left differences were also observed, with higher tissue volume and pore space on the right side. **Conclusions**: These findings provide baseline reference data on the zygomatic bone’s trabecular structure in a young, systemically healthy population. Further clinical studies in patient groups more representative of typical zygomatic implant candidates, including both older individuals with age-related maxillary atrophy and younger patients with trauma- or pathology-related bone loss, are needed to confirm the relevance of these findings to implant predictability and safety.

## 1. Introduction

Zygomatic implants have been used for many years for the restoration of atrophic maxilla and repair of congenital and acquired deficiencies in the maxillary region [1]. When placing the zygomatic implant, it is placed in the trabecular part of the zygomatic bone, starting from the first molar region. Treatment of severe maxillary atrophy with zygomatic implants provides an advantage over bone grafting, where the time to prosthesis is significantly longer, by allowing immediate temporary prosthesis within 24 h after surgery [2,3]. However, they are longer than traditional intrabone implants and require technical precision as they are applied to areas where surgical vision is limited [4]. In addition, since incorrect angles may pose a risk of perforation in the infratemporal fossa or orbital floor, grafting is still considered the gold standard today [2,5]. In reconstructive oral and maxillofacial surgery, bone grafts are commonly harvested from the ileum, ribs, calvaria, and intraoral sites. The use of local donor areas offers significant advantages, including easier surgical access, reduced anesthesia duration, and shorter operation time. Among intraoral donor sites, the mandibular ramus is frequently selected due to its lower risk of postoperative complications [6,7]. In order for the implants placed by these two methods to be considered successful, implant stability must be ensured [8].

Primary stability is the stability seen as a result of the mechanical grip of the surrounding bone tissue at the moment the implant is placed. It is stated that it is necessary for the osseointegration of the implant and its long-term success [9,10]. The higher the primary stability, the less mobility of the implant relative to the surrounding bone. Thus, direct bone healing and osseointegration can occur, and the implant survival rate increases. The main factors affecting primary stability are implant design, bone condition, and surgical techniques [11]. Dental implants are in contact with the trabecular part of the bone, so examining the microarchitecture of the trabecular bone before the implant is an important component [12].

Cone beam computed tomography (CBCT) is frequently used to evaluate changes in trabecular bone microarchitecture [13]. CBCT offers many advantages such as lower radiation doses, shorter acquisition times, high resolution, and affordability [12].

This study aims to evaluate and compare trabecular bone microarchitectural features with CBCT, focusing on anatomical points important in zygomatic implants and the angle of mandible, a representative measurement site within the mandibular ramus donor region.

## 2. Materials and Methods

This cross-sectional study was conducted retrospectively on CBCT images retrieved from the archive of Ankara University Faculty of Dentistry, Department of Dentomaxillofacial Radiology. Archived CBCT examinations performed between 24 September 2014 and 24 March 2022 were considered for inclusion. Ethical approval was obtained through the Near East University Faculty of Dentistry Ethics Committee review board, in accordance with the collaborative arrangement between the study institutions, which approved the study protocol (protocol number YDU/2024/126-1869). All the study participants provided written informed consent. A total of 847 CBCT scans of patients aged 20 to 30 years were initially screened; after applying the inclusion and exclusion criteria below, 99 patients (48 males, 51 females) were included in the final study sample. Of the 748 excluded scans, 628 were excluded because the anatomical regions required for this study were not fully captured within the field of view, 68 were excluded on the basis of the pathological (cystic or tumoral), post-surgical (prior surgery in the regions of interest), or syndromic craniofacial exclusion criteria described below, and 52 were excluded because of a missing or atypically formed mandibular first molar tooth. An a priori sample size calculation was performed in R (version 4.3.3; R Core Team, Vienna, Austria), based on the noncentral F-distribution for a one-way repeated-measures ANOVA with four repeated conditions (the four anatomical regions used for the region comparisons), assuming a conservative effect size (Cohen’s f = 0.17), α = 0.05, and power = 0.80; this indicated that a minimum of 96 patients would be required, and the final sample of 99 patients satisfied this requirement. Eligible CBCT scans were reviewed sequentially and had originally been acquired for a range of clinical indications, most commonly evaluation of third molar (wisdom tooth) position and dental implant treatment planning; a scan was selected for inclusion when all anatomical regions required for this study were fully captured within the field of view, irrespective of the original indication for imaging. Each included patient contributed a single CBCT scan, and scan eligibility and selection were performed jointly by two experienced examiners (E.K. and I.O.).

### 2.1. Inclusion Criteria

Systemically healthy male and female patients aged 20–30 years. CBCT scans were captured with a Planmeca ProMax 3D Max Pro Face model (Helsinki, Finland), 23.0 × 17.0 cm field of view (FoV), with exposure settings of 96 kV, 10 mA, and 0.4 mm voxel size. All CBCT examinations included in this study were acquired using this same device and a standardized imaging protocol throughout the study period.

### 2.2. Exclusion Criteria

Patients with cysts or tumor pathologies in the bone in the area where the measurements were made, patients who underwent surgery in the area where the measurements were made, and syndromic cases that systemically affected the craniofacial region were not included. Patients with a missing or atypically formed mandibular first molar tooth in the region of interest were also excluded.

**Figure 1:**
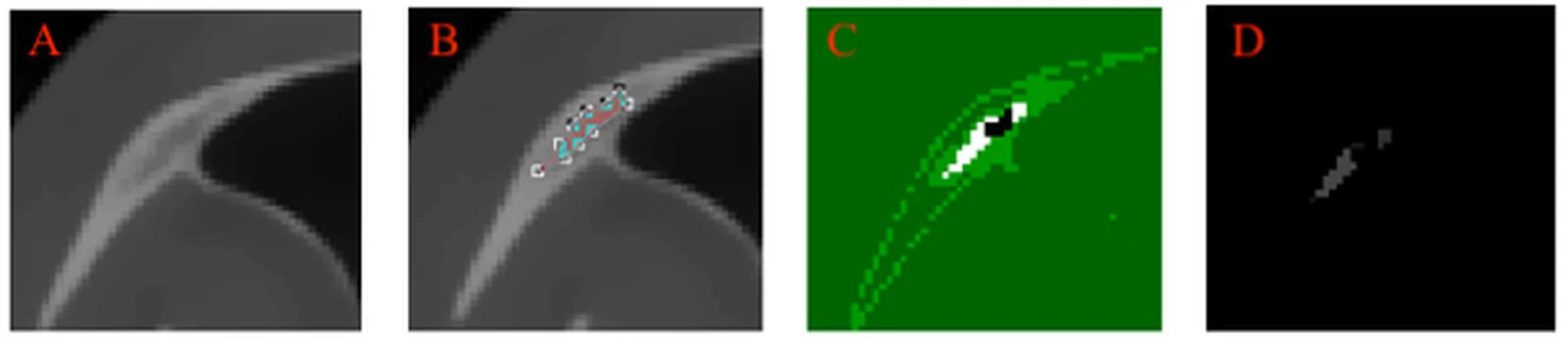
Representative workflow for trabecular bone analysis at the zygomatic bone. (**A**) Raw image; (**B**) region of interest selection; (**C**) binary segmentation; (**D**) morphometric analysis output.

**Figure 2:**
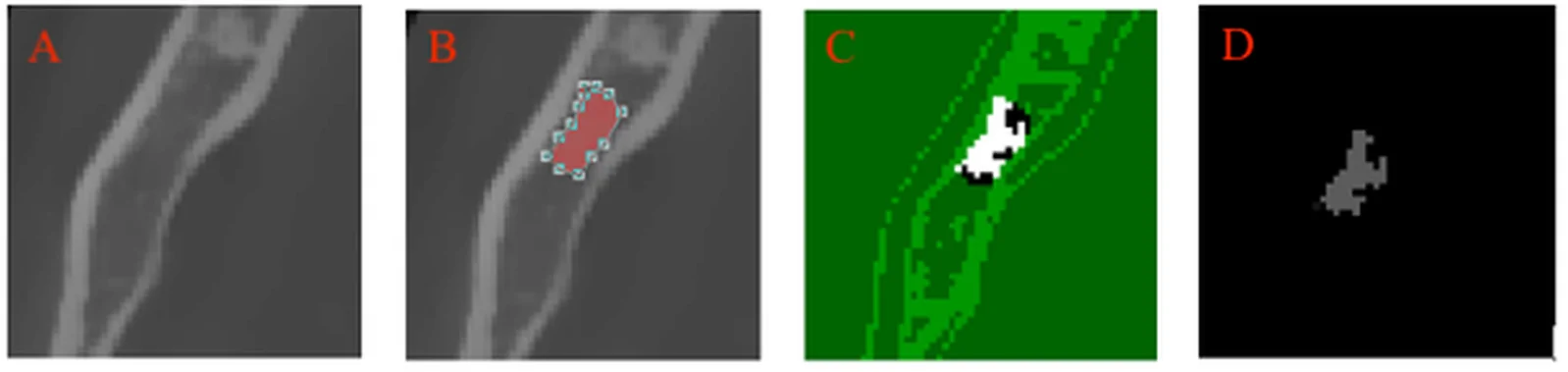
Representative workflow for trabecular bone analysis at the first molar region. (**A**) Raw image; (**B**) region of interest selection; (**C**) binary segmentation; (**D**) morphometric analysis output.

**Figure 3:**
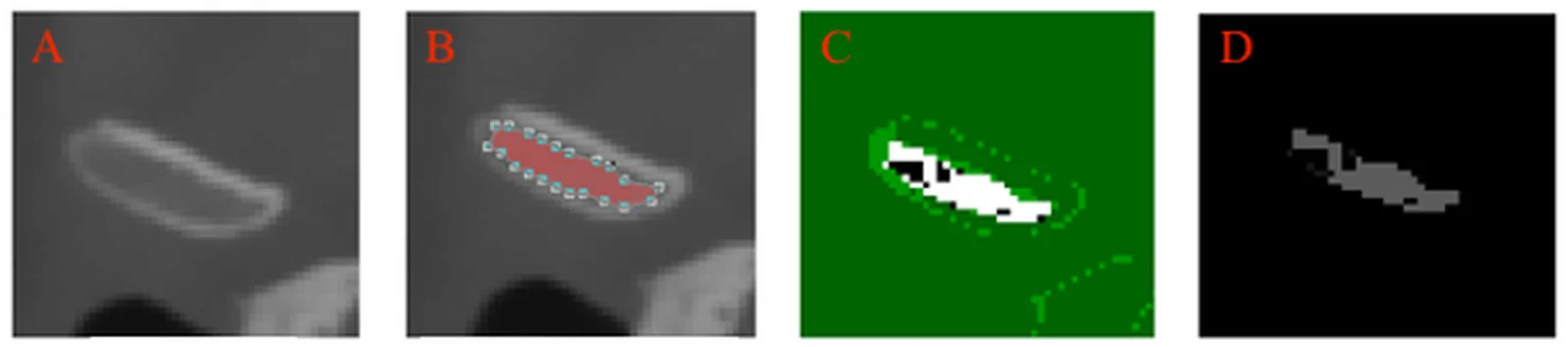
Representative workflow for trabecular bone analysis at the mandibular condyle. (**A**) Raw image; (**B**) region of interest selection; (**C**) binary segmentation; (**D**) morphometric analysis output.

**Figure 4:**
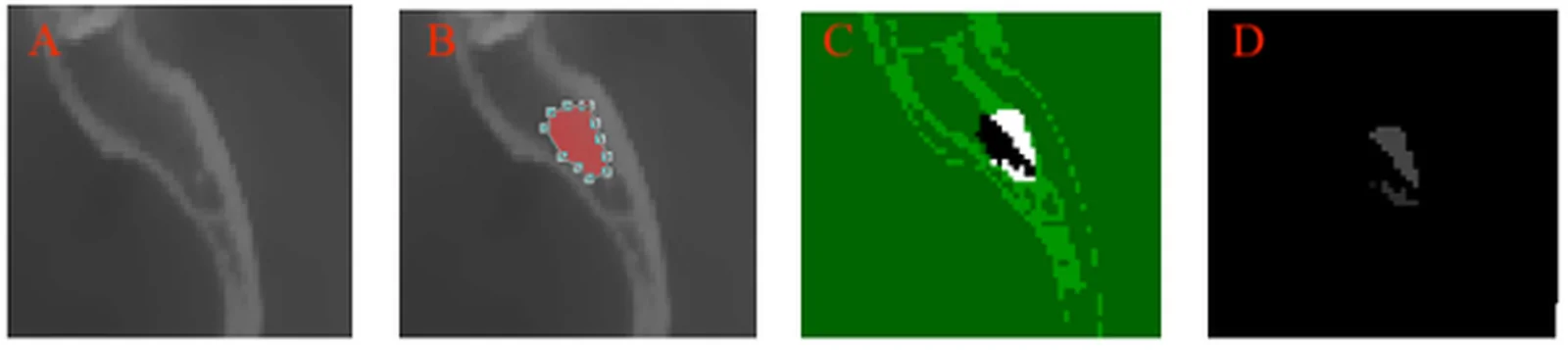
Representative workflow for trabecular bone analysis at the angle of the mandible. (**A**) Raw image; (**B**) region of interest selection; (**C**) binary segmentation; (**D**) morphometric analysis output.

Zygomatic bone, first molar tooth region, mandibular condyle, and angle of mandible were evaluated with CBCT. The first molar region, mandibular condyle, and angle of mandible were selected as comparator sites because they represent anatomically consistent, readily identifiable landmarks that can be reliably delineated on CBCT across patients; in addition, the mandibular ramus region, represented here by the angle of mandible, is an established intraoral donor site for autogenous bone grafting, allowing the zygomatic trabecular structure to be compared with a region of recognized surgical relevance, the first molar region corresponds to the starting point of the zygomatic implant trajectory, and the mandibular condyle was included as a comparator site subjected to a functional loading environment distinct from masticatory occlusal forces (temporomandibular joint biomechanics), providing an additional physiological reference point against which the trabecular architecture of the implant-related sites could be contrasted. The first molar region was defined as the trabecular bone extending from the apex of the mandibular first molar tooth to the adjacent cortical (basal) bone boundary. Volume images of the patients were transferred to CTAn software (version 1.18.4.0+, Bruker microCT, Kontich, Belgium) to evaluate the trabecular bone microstructure. For each patient, a two-dimensional region of interest (ROI) was manually delineated on each individual axial slice using the polygon selection tool, with the largest possible area that could be placed entirely within the trabecular bone without extending into the cortical boundary selected on each slice, as illustrated for a single representative slice in Figure 1, Figure 2, Figure 3 and Figure 4; the resulting volume of interest (VOI) dimensions therefore varied between anatomical regions according to local bone morphology; for a given anatomical region, the same anatomical landmarks and equivalent starting and ending slice levels were used consistently across patients and between the right and left sides. Because absolute VOI size differed among the four regions, both size-dependent (absolute) parameters, namely tissue volume, bone volume, and total pore volume, and size-independent (relative) parameters, namely percent bone volume (BV/TV), structure model index, trabecular thickness, trabecular number, trabecular separation, fractal dimension, and total porosity percentage, were reported and analyzed in parallel, allowing regional comparisons of trabecular microarchitecture to be interpreted independently of the absolute VOI size used at each site. The number of consecutive axial slices included in the analysis varied according to anatomical size, averaging approximately 15 for the zygomatic bone, 15 for the first molar region, 25 for the mandibular condyle, and 15 for the angle of mandible. The ROIs delineated across consecutive slices were merged through interpolation to obtain the three-dimensional VOI analyzed for each region. All measurements were performed by a single examiner (E.K.), who received calibration training under the supervision of a senior investigator (K.O.) at the start of the study. The following 3D microstructural parameters were calculated for each patient: tissue volume (TV); bone volume (BV); percent bone volume (BV/TV, the ratio of the segmented trabecular bone volume to the total volume of the trabecular volume of interest); structure model index (SMI, an indicator of trabecular structure, equal to 0 for parallel plates and 3 for cylindrical rods); trabecular thickness (Tb.Th, mean thickness of trabeculae); trabecular number (Tb.N, average number of trabeculae per millimeter); trabecular separation (Tb.Sp, mean distance between trabeculae); fractal dimension; total volume of pore space; and total porosity percentage.

All parameters listed above except fractal dimension were calculated automatically by the CTAn software’s 3D morphometric analysis module using standard stereological algorithms applied to the binarized VOI. To distinguish trabecular bone from marrow spaces, all images were binarized using a fixed gray-value threshold range of 80–115. This threshold range was determined empirically by testing multiple candidate ranges on representative images from each region and selecting the range that produced the clearest binary segmentation of trabecular bone across all four anatomical regions upon visual inspection; a formal quantitative sensitivity analysis of threshold selection was not performed. Representative examples of the raw image, region of interest selection, binary segmentation, and resulting morphometric analysis for each anatomical region are shown in Figure 1, Figure 2, Figure 3 and Figure 4. Fractal dimension was calculated using the Kolmogorov (box-counting) method available in the “3D Analysis” option of the morphometry module of the CTAn software, in which the volume was divided into cubes of equal size and the number of cubes containing a portion of the object’s surface was counted; this process was repeated for cubes of different edge lengths. The three-dimensional fractal dimension (3D-FD) was calculated from the slope of the log-log plot of cube count versus cube edge length. To assess intraobserver reliability, measurements for all parameters were repeated after two weeks in 20% of cases.

Statistical analyses were performed using Python (version 3.13.16; Python Software Foundation, Wilmington, DE, USA) with the scipy.stats library (version 1.18.1). Normality of distribution was assessed using the Shapiro–Wilk test on the raw, region-specific values for each parameter to determine whether the repeated-measures ANOVA or the Friedman test was the more appropriate omnibus test for the region comparisons described below; a separate test of residual normality, which is the assumption formally required by repeated-measures ANOVA, was not performed, and this is noted as a limitation for the one parameter (trabecular number, Tb.N) for which the repeated-measures ANOVA criterion was met. Because the four anatomical regions (zygoma, condyle, angle of mandible, and molar) were measured within the same individuals, comparisons among regions were treated as repeated measures: one-way repeated-measures ANOVA was used for normally distributed variables, and the Friedman test was used otherwise. When the omnibus test was significant, pairwise comparisons were performed using the Wilcoxon signed-rank test, with the Holm-Bonferroni correction applied to control for multiple comparisons. For the parameter for which repeated-measures ANOVA served as the omnibus test (trabecular number, Tb.N), Mauchly’s test was used to assess the assumption of sphericity, which was violated; the Greenhouse-Geisser-corrected omnibus result remained significant. For the pairwise post hoc comparisons, the Wilcoxon signed-rank test was used uniformly across all ten parameters, including Tb.N, rather than switching to the paired t-test for this one parameter. This choice was made for methodological consistency, since normality was assessed only at the omnibus level and not separately for each of the six pairwise within-subject difference distributions generated per parameter; the Wilcoxon signed-rank test does not require this additional, untested assumption and entails only a small loss of statistical power relative to the paired t-test under normality (asymptotic relative efficiency ≈ 0.955). For sex and side comparisons, region-level pseudoreplication was avoided by first aggregating each patient’s measurements to a single value per comparison group: for sex comparisons, the eight measurements per patient (four regions × two sides) were averaged into one overall value per patient; for right–left comparisons, the four regional values on each side were averaged to yield one left-side and one right-side value per patient. This patient-level aggregation approach was chosen over a mixed-effects modeling framework incorporating region, sex, side, and their interactions as fixed effects with patient as a random effect, in order to keep the statistical framework consistent with the non-parametric, distribution-free methods used elsewhere in this study and to avoid the additional distributional and model-specification assumptions that a mixed-effects approach would introduce given the moderate sample size. The resulting loss of region-specific resolution for the sex and side comparisons is acknowledged as a limitation (see Limitations) and represents a direction for future region-stratified analysis, particularly for the zygoma. For comparisons between sexes (independent groups), the independent-samples t-test was used for normally distributed variables with homogeneous variances (Levene’s test), Welch’s t-test was used when variances were unequal, and the Mann–Whitney U test was used for non-normally distributed variables. For right–left side comparisons, given the paired data structure, the paired-samples t-test or Wilcoxon signed-rank test was applied according to the normality of the difference scores. The relationship between age and each parameter was examined using Pearson’s or Spearman’s correlation coefficient, depending on distribution. Holm-Bonferroni correction for multiple comparisons was also applied within each of the sex-comparison, side-comparison, and age-correlation families (ten parameters each), analogous to its use for the region-pairwise comparisons described above. The significance level was set at α = 0.05. Intraobserver reliability was assessed using the intraclass correlation coefficient (ICC), and 95% confidence intervals were calculated for each ICC estimate (Table 1).

During preparation of this manuscript, a generative artificial intelligence tool (Claude, Anthropic; Claude Sonnet model family) was used to assist with correcting language and grammar. The tool was not used for study design, data collection, statistical analysis, or interpretation of results; all AI-assisted output was reviewed, verified, and edited by the authors, who take full responsibility for the content of this publication.

## 3. Results

A total of 99 patients (48 males [48.5%] and 51 females [51.5%]) were included in the study. The mean age of patients was 23.4 years.

Mean and standard deviation values for trabecular bone structural parameters are shown in Table 2.

Trabecular bone microarchitectural parameters differed significantly across the four anatomical regions for every parameter examined (Table 3). Because the four regions were measured within the same patients, comparisons were treated as repeated measures and analyzed with one-way repeated-measures ANOVA (for normally distributed variables) or the Friedman test (otherwise), followed by Wilcoxon signed-rank pairwise comparisons with Holm-Bonferroni correction. Tissue volume (TV) differed significantly among regions (Friedman χ^2^(3) = 164.93, *p* < 0.001, Kendall’s W = 0.555), with the lowest mean value in the condyle (1562.58 ± 534.91 mm^3^) and the highest in the zygoma (3596.05 ± 1304.20 mm^3^). Bone volume (BV) also differed significantly (Friedman χ^2^(3) = 74.39, *p* < 0.001, Kendall’s W = 0.250), lowest in the condyle (497.91 ± 236.46 mm^3^) and highest in the zygoma (807.54 ± 334.08 mm^3^). Percent bone volume (BV/TV) differed significantly among regions (Friedman χ^2^(3) = 74.04, *p* < 0.001, Kendall’s W = 0.249), with the lowest value in the zygoma (22.85 ± 6.14%) and the highest in the condyle (32.44 ± 10.52%); values in the angle of mandible (32.33 ± 7.14%) and molar region (28.96 ± 9.36%) were intermediate. The structure model index (SMI) differed significantly among regions (Friedman χ^2^(3) = 78.04, *p* < 0.001, Kendall’s W = 0.263), with the lowest value in the angle of mandible (0.57 ± 0.53) and the highest in the condyle (1.21 ± 0.54). Trabecular thickness (Tb.Th) differed significantly among regions (Friedman χ^2^(3) = 42.72, *p* < 0.001, Kendall’s W = 0.144), lowest in the molar region (1.42 ± 0.36 mm) and highest in the zygoma (1.69 ± 0.33 mm). Trabecular number (Tb.N) differed significantly among regions (repeated-measures ANOVA F(3,294) = 100.91, *p* < 0.001, partial η^2^ = 0.507), lowest in the zygoma (0.14 ± 0.03 1/mm) and highest in the angle of mandible (0.22 ± 0.04 1/mm). Mauchly’s test indicated that the sphericity assumption was violated for this comparison (W = 0.761, χ^2^(5) = 26.46, *p* < 0.001); the Greenhouse-Geisser-corrected result remained highly significant (ε = 0.841, *p* < 0.001), confirming the omnibus finding. Trabecular separation (Tb.Sp) differed significantly among regions (Friedman χ^2^(3) = 87.16, *p* < 0.001, Kendall’s W = 0.293), lowest in the angle of mandible (2.18 ± 0.56 mm) and highest in the zygoma (3.30 ± 0.78 mm). Fractal dimension (FD) differed significantly among regions (Friedman χ^2^(3) = 121.27, *p* < 0.001, Kendall’s W = 0.408), lowest in the zygoma (1.90 ± 0.09) and highest in the molar region (2.08 ± 0.28). Total pore volume (Po.V.tot) differed significantly among regions (Friedman χ^2^(3) = 166.76, *p* < 0.001, Kendall’s W = 0.561), lowest in the condyle (1064.68 ± 422.25 mm^3^) and highest in the zygoma (2785.74 ± 1076.49 mm^3^). Total porosity percentage (Po.tot) differed significantly among regions (Friedman χ^2^(3) = 74.04, *p* < 0.001, Kendall’s W = 0.249), lowest in the condyle (67.56 ± 10.52%) and highest in the zygoma (77.15 ± 6.14%). Holm-corrected pairwise comparisons for each parameter are reported in Table 4, with corresponding effect sizes (rank-biserial correlation, r) and 95% confidence intervals reported in Table 5.

Right–left side comparisons (pooled across all four regions per patient, *n* = 99) were also performed using the paired-samples t-test or Wilcoxon signed-rank test according to the normality of the difference scores, with Holm-Bonferroni correction applied across the ten parameters tested. Tissue volume was significantly higher on the right side than the left (Wilcoxon signed-rank, W = 1045, *p* < 0.001, Holm-adjusted *p* < 0.01, r = 0.33 [0.14, 0.50]; right 2293.06 ± 626.77 mm^3^ vs. left 2120.66 ± 582.37 mm^3^). Total pore volume was likewise significantly higher on the right (Wilcoxon signed-rank, W = 1023, *p* < 0.001, Holm-adjusted *p* < 0.01, r = 0.33 [0.14, 0.50]; right 1675.01 ± 479.65 mm^3^ vs. left 1543.90 ± 460.32 mm^3^). Trabecular number was significantly higher on the right side (paired t-test, t(98) = −2.84, *p* = 0.006, Holm-adjusted *p* = 0.048, dz = −0.29 [−0.49, −0.08]; right 0.19 ± 0.03 1/mm vs. left 0.19 ± 0.02 1/mm), and bone volume was significantly higher on the right (Wilcoxon signed-rank, W = 1705, *p* = 0.007, Holm-adjusted *p* = 0.049, r = 0.27 [0.08, 0.45]; right 618.05 ± 184.45 mm^3^ vs. left 580.69 ± 161.81 mm^3^); given how narrowly these last two results survived correction, they should be interpreted with caution. Trabecular thickness was nominally higher on the left side before correction (Wilcoxon signed-rank, W = 1812, *p* = 0.021; left 1.56 ± 0.21 mm vs. right 1.52 ± 0.20 mm), but this difference did not remain significant after Holm-Bonferroni correction (Holm-adjusted *p* = 0.126) and is therefore reported as inconclusive rather than a confirmed side difference. No significant right–left differences were found for percent bone volume (*p* = 0.373), structure model index (*p* = 0.067), trabecular separation (*p* = 0.469), fractal dimension (*p* = 0.145), or total porosity percentage (*p* = 0.373).

Comparisons between sexes (pooled across regions), with Holm-Bonferroni correction applied across the ten parameters tested, showed significantly higher tissue volume in males than females (Welch’s t-test, t = 5.59, *p* < 0.001, Holm-adjusted *p* < 0.01, Cohen’s d = 1.14 [0.71, 1.57]; male 2498.89 ± 588.53 mm^3^ vs. female 1932.53 ± 394.75 mm^3^), as well as significantly higher bone volume (Welch’s t-test, t = 4.78, *p* < 0.001, Holm-adjusted *p* < 0.01, Cohen’s d = 0.97 [0.55, 1.39]), total pore volume (Welch’s t-test, t = 5.45, *p* < 0.001, Holm-adjusted *p* < 0.01, Cohen’s d = 1.11 [0.69, 1.53]), and trabecular separation (Mann–Whitney U = 1654, *p* = 0.003, Holm-adjusted *p* = 0.021, r = −0.35 [−0.51, −0.16]) in males; all four of these differences remained significant after correction. No significant sex differences were found for percent bone volume (*p* = 0.919), structure model index (*p* = 0.986), trabecular thickness (*p* = 0.447), trabecular number (*p* = 0.730), fractal dimension (*p* = 0.947), or total porosity percentage (*p* = 0.919).

No significant correlation was found between age and any of the trabecular bone microarchitectural parameters examined (Spearman’s correlation, all *p* > 0.05; TV: r = −0.130, 95% CI [−0.319, 0.069], *p* = 0.201; BV: r = −0.079, 95% CI [−0.272, 0.120], *p* = 0.438; BV/TV: r = 0.034, 95% CI [−0.165, 0.230], *p* = 0.740; SMI: r = 0.086, 95% CI [−0.113, 0.279], *p* = 0.397; Tb.Th: r = 0.021, 95% CI [−0.177, 0.218], *p* = 0.838; Tb.N: r = 0.067, 95% CI [−0.132, 0.261], *p* = 0.510; Tb.Sp: r = 0.047, 95% CI [−0.152, 0.242], *p* = 0.644; FD: r = −0.065, 95% CI [−0.259, 0.134], *p* = 0.524; Po.V.tot: r = −0.124, 95% CI [−0.314, 0.075], *p* = 0.222; Po.tot: r = −0.034, 95% CI [−0.230, 0.165], *p* = 0.740; 95% confidence intervals were computed from the Fisher z-transformation). Holm-Bonferroni correction across these ten correlations did not alter these conclusions, as none reached nominal significance even before adjustment.

## 4. Discussion

Primary stability is the stability seen as a result of the mechanical grip of the surrounding bone tissue at the moment the implant is placed [10]. Typically, trabecular bone and cortical bone in the peri-implant area seriously affect the primary stability of the dental implant, which is a critical factor affecting the survival rate of the implant. Since implants are placed in trabecular bone, trabecular bone quality is especially important [14]. It should also be emphasized that primary implant stability is influenced not only by trabecular bone microarchitecture but also by cortical bone engagement, implant design, surgical trajectory, and insertion technique; the present study characterizes only the trabecular component and does not account for these additional biomechanical determinants of stability. It should be noted that bone quality is a broader concept than the trabecular microarchitectural parameters assessed in this study; it also encompasses mineral content, structural organization, and mechanical properties of bone tissue, of which trabecular microarchitecture is only one component, and the parameters reported here (tissue and bone volume, percent bone volume, trabecular thickness, number and separation, and fractal dimension) characterize this structural component rather than bone mineral density or mechanical strength directly. The quality of bone is not only related to its mineral content or quantity but also to its structure. A well-structured thin trabecular bone may indicate a highly vascularized area, which may support faster bone regeneration around the implant [15]. However, the relationship between the microarchitectural parameters reported here and actual implant primary stability was not directly tested in this study. Therefore, knowing the trabecular patterns in the implant area well before surgery will help the clinician in choosing the appropriate implant protocol.

Micro-CT is considered the gold standard method for trabecular microstructure evaluation [16]. Although micro-CT has been adopted as the standard for the analysis of trabecular bone structures, it is not suitable for use in clinical diagnoses due to scanning range limitations [12]. In addition to its limited scanning range, micro-CT delivers substantially higher radiation doses than CBCT, which restricts its clinical use in living patients to ex vivo or cadaveric specimens; CBCT was therefore used in the present in vivo study as a clinically applicable, lower-dose alternative. In recent years, many researchers have used dental CBCT as a method of assessing bone density prior to dental implant surgery. Studies have suggested that trabecular bone parameters evaluated with CBCT show a strong correlation with micro-CT and that trabecular bone parameters can be evaluated with CBCT. This allows the clinician to estimate initial implant stability and the likelihood of immediate or early loading using CBCT scans prior to implant placement [16,17,18,19]. We could not find any study in the literature comparing trabecular parameters of different bone regions with CBCT. Therefore, to our knowledge, this study provides one of the first comparative evaluations of trabecular bone structure across these regions using CBCT in a relatively large sample.

Bone grafting is the most preferred method in the rehabilitation of atrophic maxilla [5]. Zygomatic implants were proposed by Brånemark et al. to rehabilitate the atrophic maxilla and reconstruct congenital and acquired maxilla defects and have been used for many years [20,21]. The most recent systematic review reports a 12-year cumulative survival rate of 95.21% based on 68 studies with 4556 zygomatic implants in 2161 patients and concludes that the technique has high predictability with good clinical outcomes [22]. However, data on zygomatic bone quality are still insufficient, especially when compared to other bone regions. For this reason, it is not preferred much. There are not many studies in the literature examining the trabecular part of the zygomatic bone; the available studies have evaluated it using micro-CT [23,24,25,26]. In our study, the zygomatic trabecular bone evaluated by CBCT had significantly higher tissue and bone volume than the other three regions examined (Table 2 and Table 4). Because VOI dimensions were defined, at each anatomical site, as the largest volume that could be placed entirely within the trabecular bone without extending into the cortical boundary (see Methods), the greater absolute VOI, tissue volume, and bone volume observed in the zygoma are expected, at least in part, to reflect the larger anatomical extent of trabecular bone available at this site rather than solely a difference in true trabecular bone volume independent of anatomy; the relative parameters reported in parallel, most notably BV/TV, are therefore the more appropriate basis for comparing trabecular bone volume fraction among the four regions independent of this anatomical size difference. These results were consistent with the study conducted by Bertl et al. in 2015 [23]. They suggested that the zygomatic bone has trabecular bone quality equivalent to the trabecular bone quality of the anterior maxilla, and therefore they said that zygomatic implants are a good option in atrophic maxilla [23]. This greater tissue and bone volume should not, however, be interpreted as indicating superior trabecular bone quality at the zygomatic site: the zygoma simultaneously exhibited the lowest percent bone volume and trabecular number and the greatest trabecular separation among the four regions studied, indicating a comparatively sparse, less densely interconnected trabecular network within a larger overall volume. Bone quantity and trabecular microarchitectural quality should therefore be regarded as distinct, and in this case divergent, characteristics of the zygomatic bone. Many studies have reported that the percent bone volume (BV/TV) ratio is the most important parameter in determining bone quality. As a result of optimal bone adaptation to masticatory forces, increased BV/TV has been found in areas where large masticatory forces are received [16]. It should be noted that total porosity percentage (Po.tot) is mathematically complementary to BV/TV (Po.tot = 100% − BV/TV) and is therefore not statistically independent of it; the two parameters are reported together for completeness and consistency with the prior literature, but their regional differences should be interpreted as reflecting the same underlying bone volume fraction rather than as independent findings. In studies conducted in the literature, the BV/TV ratio in the mandible varies between 9.24% and 43.74% [27,28]. In our study, the highest BV/TV ratio was found in the mandibular condyle, followed by the angle of mandible and molar region, with the lowest value in the zygomatic bone (Table 2). Parsa et al. attributed this type of difference in the literature to differences in the scanning system used [16].

Structure model index, trabecular thickness, trabecular number, and trabecular separation are important parameters that show bone quality. Additionally, the structure model index is an important indicator of osteoporotic deterioration of trabecular bone. The higher the trabecular number, the more compact the bone is. In studies conducted in the literature, mandibular trabecular number values vary between 0.007 and 2.19 mm^−1^ [28]. In our study, the lowest trabecular number value was found in the zygomatic bone. On the contrary, trabecular thickness and trabecular separation were highest in the zygomatic bone.

In dental radiology, fractal dimension calculation has been used to identify bone changes associated with periapical periodontitis, periodontal diseases, bone surgery, and systemic diseases, so it can be used to evaluate trabecular bone structure [29]. In their study, Lee et al. showed that there was a statistically significant relationship between the primary stability of the implant and the fractal dimension [30]. In our study, the lowest fractal dimension values were found in the zygomatic bone, while the highest values were found in the molar region. As with the other microarchitectural parameters reported here, the relationship between fractal dimension and actual implant primary stability was not directly tested in the present study. Three-dimensional fractal dimension has been more extensively validated using micro-CT; its behavior when calculated from CBCT images at the 0.4 mm voxel size used here has not been separately validated against micro-CT-derived values, and this should be considered when interpreting the absolute FD values reported.

In addition to regional differences, significant sex-based and right–left variations were observed in the present study. Tissue volume, bone volume, trabecular separation, and total pore volume were significantly higher in males than in females, whereas no significant sex differences were found for percent bone volume, structure model index, trabecular thickness, trabecular number, fractal dimension, or total porosity. This pattern is consistent with well-documented craniofacial sexual dimorphism, whereby males generally present with larger overall skeletal dimensions than females [31]. The absence of a corresponding difference in relative measures such as BV/TV and SMI suggests that, despite this size difference, the underlying quality and organization of trabecular bone may be broadly comparable between sexes in this young, healthy population. This finding should nonetheless be interpreted with some caution, as other CBCT-based studies of the mandible have reported sex-related differences in fractal dimension and bone volume fraction [32], indicating that the presence and direction of sex differences in trabecular microarchitecture may vary according to the specific anatomical site and parameter examined.

Right–left comparisons, pooled across the four regions, revealed significantly higher tissue volume and total pore volume on the right side after correction for multiple comparisons, with bone volume and trabecular number also higher on the right, though these latter two differences survived correction only narrowly and should be interpreted with caution; no side differences were observed for percent bone volume, structure model index, trabecular separation, fractal dimension, or total porosity, and the nominal difference in trabecular thickness did not survive Holm-Bonferroni correction. A tendency toward right-sided skeletal dominance has previously been reported in the craniofacial complex, including at the zygomatic point specifically [33], as well as in the mandibular ramus, where the right side has been found to be longer than the left in both sexes [34]. The right–left differences observed here for absolute volumetric parameters, without corresponding differences in relative bone quality parameters such as BV/TV, may therefore reflect this broader pattern of skeletal asymmetry rather than a clinically meaningful difference in bone quality between sides. From a clinical standpoint, these sex- and side-related findings suggest that surgeons should anticipate a degree of normal anatomical variation in bone volume between sexes and sides when planning zygomatic implant placement, even though the relative quality of the trabecular bone appears comparable.

This study has some limitations that should be acknowledged. The sample was restricted to systemically healthy patients aged 20–30 years; this age distribution reflects the retrospective nature of the study, as full craniofacial CBCT scans meeting the inclusion criteria were predominantly available from younger patients in the departmental archive, and it allowed trabecular bone parameters to be assessed under physiological conditions without the confounding effects of age-related bone loss. It should also be noted that zygomatic implants are not indicated exclusively in older, edentulous patients with severe maxillary atrophy; they are also applied in relatively younger patients presenting with extensive bone loss secondary to trauma, tumor resection, or other etiologies. Nonetheless, the present sample should be regarded as a young, systemically healthy reference population rather than one fully representative of the broader range of zygomatic implant candidates, particularly older edentulous patients with age-related bone loss. In addition, the retrospective design and the exclusion of patients with cystic, tumoral, or post-surgical changes ensured a homogeneous dataset for characterizing normal trabecular architecture, but these criteria mean that the results should be regarded as baseline reference values rather than a direct description of bone quality in markedly atrophic or previously operated maxillae. Finally, although the zygomatic bone was compared with several established mandibular reference sites, maxillary anatomical regions directly involved in implant-supported maxillary rehabilitation were not included in the present comparison. Additionally, CBCT gray-scale values were not calibrated against a density phantom to derive Hounsfield-equivalent units; because all scans were acquired on the same device using identical exposure parameters, this is unlikely to have affected the relative comparisons among regions within this study, but it limits direct comparability of absolute density-related values with studies using different CBCT systems or medical CT. Furthermore, images were acquired with a 0.4 mm voxel size, at which partial volume effects can lead to overestimation of trabecular thickness and underestimation of trabecular number and separation relative to micro-CT, which offers substantially higher spatial resolution; because this resolution was applied uniformly across all four regions, it is unlikely to have biased the relative comparisons among them, but the absolute parameter values reported here should be interpreted with this resolution constraint in mind. Sex- and side-based comparisons were performed after pooling measurements across all four anatomical regions per patient to avoid region-level pseudoreplication (see Methods); as a result, this study cannot determine whether the observed sex- and side-related differences are uniform across regions or are driven disproportionately by one or more specific sites, including the zygoma. Region-specific sex and side comparisons, particularly for the zygoma, the region most directly relevant to zygomatic implant placement, represent an important direction for future study. Finally, normality for the repeated-measures ANOVA (applied only to trabecular number) was assessed on the raw regional values rather than on the model residuals, which is the assumption formally required by this test; this is noted as a limitation of the statistical methodology.

Future studies would benefit from extending this evaluation to older patient cohorts, including those with severe maxillary atrophy or maxillary defects secondary to tumor resection, in order to determine whether the trabecular patterns identified here are maintained across a wider age range and in more clinically representative populations. Comparative analyses including maxillary reference sites, as well as sex-stratified evaluations, would further enhance the clinical relevance of these findings. In addition, correlating CBCT-derived microarchitectural parameters with established bone density classifications, such as the Misch D1–D4 system, and with implant primary stability measures (e.g., resonance frequency analysis or insertion torque), would help translate the present reference data into more direct clinical guidance for zygomatic implant planning. Cadaveric or other ex vivo micro-CT studies could additionally provide direct micro-CT validation of the CBCT-derived trabecular parameters reported here, including at the zygomatic site, without the radiation-dose constraints that preclude micro-CT use in living patients.

## 5. Conclusions

This study demonstrated that the zygomatic bone exhibits a distinct trabecular microarchitecture compared with the mandibular condyle, angle of mandible, and first molar region, characterized by higher tissue and bone volumes but lower BV/TV and trabecular number. These findings provide baseline reference data on zygomatic trabecular structure that may support preoperative planning for zygomatic implants. More clinical studies on the trabecular architecture of the zygomatic bone, particularly in older and more clinically representative populations, are needed to safely perform zygomatic implants in the atrophic maxilla. In addition, CBCT-based assessment of trabecular bone structure before surgery may help clinicians anticipate regional differences in bone architecture and inform treatment planning; however, the direct relationship between the microarchitectural parameters reported here and clinical implant stability outcomes was not evaluated in this study. Prospective correlation of these CBCT-derived microarchitectural parameters with established bone density classifications (e.g., the Misch D1–D4 system) and with direct implant stability measures (e.g., resonance frequency analysis, insertion torque) is needed before these findings can inform clinical decisions on zygomatic implant stability, and should be regarded as a priority for future research in this area rather than a secondary consideration.

## Figures and Tables

**Table 1:** Intraobserver reliability (ICC) results. ICC(A,1): two-way random-effects model, absolute agreement, single measures.

Variable	ICC(A,1)	95% CI
Structure model index	0.894	[0.850–0.938]
Tissue volume	0.880	[0.830–0.930]
Trabecular separation	0.878	[0.827–0.929]
Bone volume	0.857	[0.798–0.916]
Trabecular thickness	0.854	[0.794–0.914]
Fractal dimension	0.851	[0.790–0.912]
Percent bone volume	0.852	[0.792–0.912]
Total volume of pore space	0.844	[0.781–0.907]
Total porosity (%)	0.835	[0.768–0.902]
Trabecular number	0.830	[0.761–0.899]

95% confidence intervals are reported alongside the point estimates in this table.

**Table 2:** Mean and standard deviation of trabecular bone structural parameters by anatomical region (*n* = 99 patients; pixel size = 400 µm).

Parameter	Region	Mean ± SD
Tissue volume (TV), mm^3^	Zygoma	3596.05 ± 1304.20
	Condyle	1562.58 ± 534.91
	Angle of mandible	1580.30 ± 793.63
	Molar region	2090.36 ± 1025.67
Bone volume (BV), mm^3^	Zygoma	807.54 ± 334.08
	Condyle	497.91 ± 236.46
	Angle of mandible	511.01 ± 279.67
	Molar region	580.52 ± 298.81
Percent bone volume (BV/TV), %	Zygoma	22.85 ± 6.14
	Condyle	32.44 ± 10.52
	Angle of mandible	32.33 ± 7.14
	Molar region	28.96 ± 9.36
Structure model index (SMI)	Zygoma	0.82 ± 0.48
	Condyle	1.21 ± 0.54
	Angle of mandible	0.57 ± 0.53
	Molar region	0.97 ± 0.34
Trabecular thickness (Tb.Th), mm	Zygoma	1.69 ± 0.33
	Condyle	1.52 ± 0.34
	Angle of mandible	1.52 ± 0.31
	Molar region	1.42 ± 0.36
Trabecular number (Tb.N), 1/mm	Zygoma	0.14 ± 0.03
	Condyle	0.21 ± 0.06
	Angle of mandible	0.22 ± 0.04
	Molar region	0.20 ± 0.05
Trabecular separation (Tb.Sp), mm	Zygoma	3.30 ± 0.78
	Condyle	2.95 ± 1.06
	Angle of mandible	2.18 ± 0.56
	Molar region	2.73 ± 0.75
Fractal dimension (FD)	Zygoma	1.90 ± 0.09
	Condyle	2.05 ± 0.11
	Angle of mandible	2.03 ± 0.13
	Molar region	2.08 ± 0.28
Total pore volume (Po.V.tot), mm^3^	Zygoma	2785.74 ± 1076.49
	Condyle	1064.68 ± 422.25
	Angle of mandible	1069.29 ± 551.34
	Molar region	1509.83 ± 825.75
Total porosity (Po.tot), %	Zygoma	77.15 ± 6.14
	Condyle	67.56 ± 10.52
	Angle of mandible	67.67 ± 7.14
	Molar region	71.04 ± 9.36

TV, tissue volume; BV, bone volume; BV/TV, percent bone volume; SMI, structure model index; Tb.Th, trabecular thickness; Tb.N, trabecular number; Tb.Sp, trabecular separation; FD, fractal dimension; Po.V.tot, total pore volume; Po.tot, total porosity percentage.

**Table 3:** Omnibus statistical comparison of trabecular bone structural parameters among the zygoma, condyle, angle of mandible, and molar regions (repeated-measures design, *n* = 99). All parameters differed significantly among regions (*p* < 0.05).

Parameter	Test	Statistic	*p* Value	Effect Size (95% CI)
Tissue volume (TV)	Friedman test	164.93	<0.001	W = 0.555 [0.503–0.602]
Bone volume (BV)	Friedman test	74.39	<0.001	W = 0.250 [0.181–0.323]
Percent bone volume (BV/TV, %)	Friedman test	74.04	<0.001	W = 0.249 [0.176–0.331]
Structure model index (SMI)	Friedman test	78.04	<0.001	W = 0.263 [0.173–0.360]
Trabecular thickness (Tb.Th)	Friedman test	42.72	<0.001	W = 0.144 [0.091–0.245]
Trabecular number (Tb.N)	Repeated-measures ANOVA	100.91	<0.001	η^2^p = 0.507 [0.457–0.573]
Trabecular separation (Tb.Sp)	Friedman test	87.16	<0.001	W = 0.293 [0.227–0.407]
Fractal dimension (FD)	Friedman test	121.27	<0.001	W = 0.408 [0.338–0.528]
Total pore volume (Po.V.tot)	Friedman test	166.76	<0.001	W = 0.561 [0.516–0.617]
Total porosity (Po.tot, %)	Friedman test	74.04	<0.001	W = 0.249 [0.181–0.334]

W, Kendall’s W (Friedman-based comparisons); η^2^p, partial eta squared (repeated-measures ANOVA). 95% confidence intervals were obtained by bootstrap resampling of patients (2000 replicates).

**Table 4:** Holm-Bonferroni-corrected pairwise comparisons (Wilcoxon signed-rank test) between anatomical regions for each trabecular bone structural parameter (*n* = 99).

Parameter	Zygoma vs. Condyle	Zygoma vs. Angle	Zygoma vs. Molar	Condyle vs. Angle	Condyle vs. Molar	Angle vs. Molar
Tissue volume (TV)	<0.001 *	<0.001 *	<0.001 *	0.783	<0.001 *	0.002 *
Bone volume (BV)	<0.001 *	<0.001 *	<0.001 *	0.645	0.048 *	0.220
Percent bone volume (BV/TV, %)	<0.001 *	<0.001 *	<0.001 *	0.892	0.029 *	<0.001 *
Structure model index (SMI)	<0.001 *	<0.001 *	0.015 *	<0.001 *	<0.001 *	<0.001 *
Trabecular thickness (Tb.Th)	<0.001 *	<0.001 *	<0.001 *	0.610	0.104	0.009 *
Trabecular number (Tb.N)	<0.001 *	<0.001 *	<0.001 *	0.761	0.272	0.035 *
Trabecular separation (Tb.Sp)	0.009 *	<0.001 *	<0.001 *	<0.001 *	0.054	<0.001 *
Fractal dimension (FD)	<0.001 *	<0.001 *	<0.001 *	0.038 *	0.004 *	<0.001 *
Total pore volume (Po.V.tot)	<0.001 *	<0.001 *	<0.001 *	0.743	<0.001 *	<0.001 *
Total porosity (Po.tot, %)	<0.001 *	<0.001 *	<0.001 *	0.892	0.029 *	<0.001 *

* *p* < 0.05, Holm-Bonferroni-corrected. Angle = angle of mandible.

**Table 5:** Effect sizes (rank-biserial correlation, r) with 95% confidence intervals for the pairwise comparisons in Table 4 (Wilcoxon signed-rank test, *n* = 99).

Parameter	Zygoma vs. Condyle	Zygoma vs. Angle	Zygoma vs. Molar	Condyle vs. Angle	Condyle vs. Molar	Angle vs. Molar
Tissue volume (TV)	0.87 [0.87–0.87]	0.85 [0.81–0.87]	0.82 [0.77–0.86]	0.04 [0.00–0.23]	0.49 [0.35–0.64]	0.30 [0.12–0.48]
Bone volume (BV)	0.76 [0.67–0.83]	0.62 [0.48–0.74]	0.58 [0.43–0.71]	0.08 [0.00–0.26]	0.25 [0.05–0.42]	0.14 [0.00–0.33]
Percent bone volume (BV/TV, %)	0.70 [0.60–0.78]	0.79 [0.72–0.83]	0.58 [0.44–0.70]	0.02 [0.00–0.21]	0.24 [0.06–0.41]	0.33 [0.14–0.50]
Structure model index (SMI)	0.58 [0.44–0.69]	0.53 [0.37–0.66]	0.23 [0.03–0.41]	0.73 [0.63–0.81]	0.41 [0.24–0.58]	0.58 [0.42–0.70]
Trabecular thickness (Tb.Th)	0.44 [0.29–0.58]	0.47 [0.30–0.61]	0.65 [0.52–0.74]	0.04 [0.00–0.25]	0.23 [0.04–0.40]	0.30 [0.11–0.48]
Trabecular number (Tb.N)	0.84 [0.81–0.86]	0.85 [0.82–0.87]	0.83 [0.79–0.86]	0.00 [0.00–0.22]	0.12 [0.00–0.30]	0.18 [0.00–0.36]
Trabecular separation (Tb.Sp)	0.30 [0.09–0.48]	0.81 [0.77–0.85]	0.55 [0.39–0.68]	0.62 [0.50–0.73]	0.22 [0.02–0.38]	0.61 [0.47–0.74]
Fractal dimension (FD)	0.79 [0.70–0.85]	0.73 [0.63–0.82]	0.67 [0.54–0.79]	0.24 [0.04–0.43]	0.33 [0.14–0.51]	0.47 [0.27–0.64]
Total pore volume (Po.V.tot)	0.87 [0.87–0.87]	0.86 [0.85–0.87]	0.83 [0.78–0.86]	0.04 [0.00–0.24]	0.49 [0.34–0.63]	0.32 [0.13–0.47]
Total porosity (Po.tot, %)	0.70 [0.61–0.79]	0.79 [0.72–0.84]	0.58 [0.43–0.70]	0.02 [0.00–0.22]	0.24 [0.08–0.41]	0.33 [0.15–0.49]

r = |Z|/√N, rank-biserial effect size; 0.1 small, 0.3 medium, 0.5 large (Cohen’s conventions). 95% CIs obtained by bootstrap resampling of patients (500 replicates). Angle = angle of mandible.

## Data Availability

The raw per-patient measurement data presented in this study are not publicly available because of patient privacy restrictions but are available from the corresponding author upon reasonable request; anonymized data may be shared for legitimate research purposes subject to institutional and ethics committee approval.
